# Shy and Bold Fish Have the Same Preference for Light Color Selection

**DOI:** 10.3390/ani14111583

**Published:** 2024-05-27

**Authors:** Qingqing Zou, Weiwei Li, Chaoshuo Zhang, Jianghui Bao, Huafei Lyu, Ming Duan

**Affiliations:** 1 State Key Laboratory of Freshwater Ecology and Biotechnology, Institute of Hydrobiology, Chinese Academy of Sciences, Wuhan 430072, China; zouqingqing@ihb.ac.cn (Q.Z.); liweiwei@ihb.ac.cn (W.L.); zhangcs@ihb.ac.cn (C.Z.); baojianghui@ihb.ac.cn (J.B.); lvhuafei@ihb.ac.cn (H.L.); 2 College of Advanced Agricultural Sciences, University of Chinese Academy of Sciences, Beijing 100101, China

**Keywords:** personality, animal welfare, phototaxis, fish behavior, *Pelteobagrus fulvidraco*

## Abstract

**Simple Summary:**

Personality and light are crucial to ensure the welfare of aquatic animals. To understand how personality affects the light color selection of fish, we tested the behaviors with different personalities under six colors of light: violet (410–420 nm), yellow (580–590 nm), green (550–560 nm), red (620–630 nm), blue (470–480 nm), and white. In this study, the yellow catfish *Pelteobagrus fulvidraco* preferred the yellow and green light over the other light colors. After identifying their preferred light colors, bold individuals reduced their frequency of exploration. The results showed that bold individuals could make a quick decision for light color selection. This study provides a reference for the welfare of juvenile yellow catfish in aquaculture.

**Abstract:**

Personality, which matters for animal welfare, demonstrates behavioral differences. Light is one of the most important factors in aquaculture. However, how fish personality affects light color selection is unclear. In this study, we tested the personality of yellow catfish *Pelteobagrus fulvidraco* juveniles and then quantified the selective behaviors of different personalities under six light colors: violet (410–420 nm), yellow (580–590 nm), green (550–560 nm), red (620–630 nm), blue (470–480 nm), and white. The results showed that juveniles preferred the yellow and green light over the other colors of light, probably due to different reasons. The average cumulative dwell time in yellow (32.81 ± 5.22%), green (21.81 ± 3.58%), and red (26.36 ± 4.89%) lights was significantly longer than the other light colors, and the average visit frequency in green light (32.00 ± 4.93%) was the most. Juveniles had the longest total moved distance in green light. Moreover, the results demonstrated that shy and bold individuals had the same preference for the green light. Bold individuals could find the preferred light colors rapidly and make quick decisions for light color selection. After identifying the preferred light colors, bold individuals reduced the frequency of exploration. This study provides a theoretical basis for the welfare of juvenile yellow catfish in aquaculture.

## 1. Introduction

Animal welfare has attracted widespread public attention and become an increasingly important academic discipline [1,2]. It refers to the well-being of animals in their surroundings, particularly their freedom and comfort [3,4]. Nowadays, concerns about animal welfare have expanded to farmed aquatic animals [5]. Changes in environmental factors can lead to stress in aquatic animals, which affects their natural behavior and reduces aquaculture production [6,7]. Fish behavior is seen as a noninvasive and an early sign of potential fish welfare problems, due to fish exhibiting abnormal behavior under stressful conditions [8]. Therefore, observing and understanding the behaviors of aquatic animals may help to improve and enhance their animal welfare.

Creating a favorable rearing environment is crucial to ensure the welfare of aquatic animals and can significantly enhance the commercial quality of aquatic products [9]. Light is a directive factor and plays a significant role in the entire life history of fishand induces fish’s metabolic system to respond appropriately [10,11,12]. How light works depends on its intensity, photoperiod, and spectrum [13]. In intensive aquaculture, artificial light, including various colors, is often used to promote fish growth and development. Recent evidence suggests that fish have different types of photoreceptors with specific visual sensitivity [14]. For example, adult zebrafish have four cone types in the red spectrum, the blue spectrum, the yellow spectrum, and the ultraviolet spectrum. The common carp *Cyprinus carpio* and the Nile tilapia *Oreochromis niloticus* showed a visual sensitivity toward the near infrared light [15]. However, knowledge of spectral types of vision is still rudimentary for most fish species. Moreover, fish respond to different lights in different ways. Fish have phototaxis, the directional movement toward (positive) or away from (negative) light sources, which helps fish access resources or avoid dangerous and unfavorable conditions [16,17]. For different light colors, fish have positive or negative phototropism varying degrees [18,19]. It is known, however, that fish’s visual system changes across different life stages, and phototactic behaviors undergo modifications [20]. Thus, fish phototactic behavior at different stages to different colors can be used as a reference for implementing aquaculture countermeasures in practical production.

Animal personality refers to the consistency of individual behavioral differences over time and across various situations [21,22,23,24]. It plays a crucial role in the life processes and adaptive capacity of animals and is of great evolutionary and ecological significance. This has made it a key focus of research in behavioral ecology [25]. Fish with different personalities show considerable individual differences in cognitive abilities [26] and habitat preferences [27]. In unpredictable environments, animals with shy or bold personalities often make decisions by a trade-off between foraging gains and the associated risk [28]. Studies of fish behavior have found that bold fish are more exploratory, move more frequently, and disperse more [29,30,31]. However, we usually think of animal welfare as a species attribute and, in fact, ignore individual differences between the same species [32]. Fish welfare assessments often involve taking samples of individuals from rearing units without taking into account the differences introduced by personality. Few studies focused on different personalities in animal welfare.

This study concentrates on *Pelteobagrus fulvidraco* (family Bagridae, genus Pelteobagrus, Richardson, 1846), which is an economically important freshwater aquaculture species in China [33]. This fish species is an omnivorous fish that is mainly carnivorous and mostly active in still water or slow-flowing rivers, with benthic life, and is very sensitive to light [34]. Previous studies have focused on the phototropism of *P. fulvidraco*, but how personality affects its choice of light colors is yet unknown. We hypothesized that the light color selection behavior of *P. fulvidraco* juveniles toward light colors is influenced by personality. To test this hypothesis, we quantified the boldness of *P. fulvidraco* juveniles and used the experimentally determined boldness and classification. Then, we compared the light color selection behaviors of bold and shy fish. This study provides insights for the welfare of juvenile yellow catfish in aquaculture and will further enrich the theoretical knowledge of behavioral ecology in the family Bagridae.

## 2. Materials and Methods

### 2.1. Maintenance of Experimental Fish

Wild-type *P. fulvidraco* juveniles were obtained from the Hubei Academy of Agricultural Sciences. Before the experiment started, a total of 24 *P. fulvidraco* juveniles were transferred to an indoor racked recirculating water culture system for a fortnight, with each tank measuring 40 cm × 30 cm × 30 cm (4 tails per tank). Experimental water quality parameters were monitored daily during the acclimation period. The water used for cultivation and experimentation was kept at 20.49 ± 0.18 °C. An air pump was used to ensure that dissolved oxygen in the water column was maintained at 6.43 ± 0.26 mg/L. Half of the water was changed daily, and the pH was 8.15 ± 0.42. The photoperiod was set to 12 h of light (on at 8:00 a.m.) and 12 h of darkness (off at 8:00 p.m.). Experimental fish were fed with frozen red worms (*Eisenia fetida*) (24 capsules/100 g) once daily at 9:00 am.

### 2.2. Experimental Design

The experiments were divided into a personality test and a light color selection experiment. To exclude the effects of feeding activity and photoperiod rhythms, each experiment was conducted at the end of feeding and sometime before the start of the dark light cycle, and the experimental period was set between 14:00 and 18:00 [35].

#### 2.2.1. Personality Test

**Figure 1:**
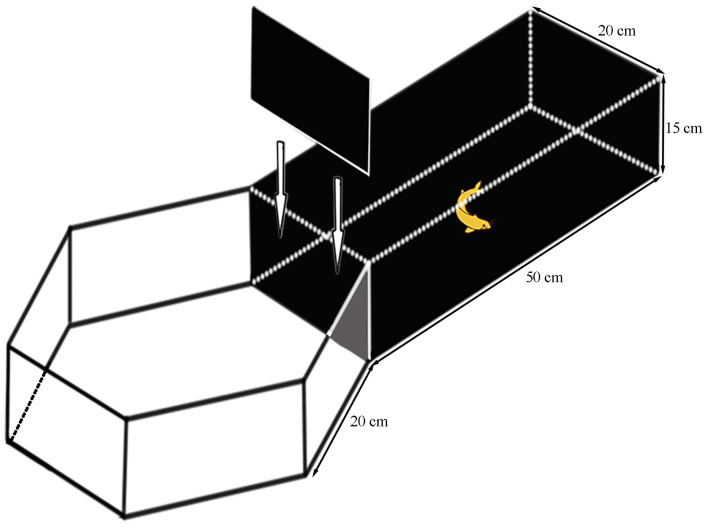
Experimental apparatus for testing fish personality.

An emergence experiment was used to conduct a shy–boldness test. Shy and bold individuals were distinguished by assessing the ability of experimental fish to leave a safe area and enter a risk area [36]. The experimental setup was constructed from acrylic and consisted of a black-covered rectangular (L × W × H = 30 cm × 20 cm × 15 cm) refuge area connected to a white square hexagonal prism (20 cm side length and 15 cm height) risk area (Figure 1) [37]. A black removable partition between the two areas was used to isolate the light from the risk area. The light intensity was measured using an HP350C spectral color illuminance meter and was approximately 600 lx in the risk zone. Fresh aeration water was used as experimental water, and the depth of the water was controlled at 7 cm. The water was changed, and the device was cleaned after each experiment. At the beginning of the experiment, a fish was randomly retrieved from the temporary recirculating water system and carefully transferred to the center of the refuge area. After 2 min of dark adaptation, video recording was started when the partition was withdrawn, and 6 min of video (30 frames/s) was recorded using a high-speed industrial camera (acA1920, 155uc NIR, Balser, Germany) located approximately 1.0 m above the risk area. At the end of the personality test, their total length and weight were measured after anesthesia with 100 mg/L MS-222. The six most shy and six most bold fish with similar size were selected from 24 *P. fulvidraco*, and their condition factor was calculated (Table 1). Each fish was isolated for three days in order to conduct follow-up experiments.

#### 2.2.2. Light Color Selection

**Figure 2:**
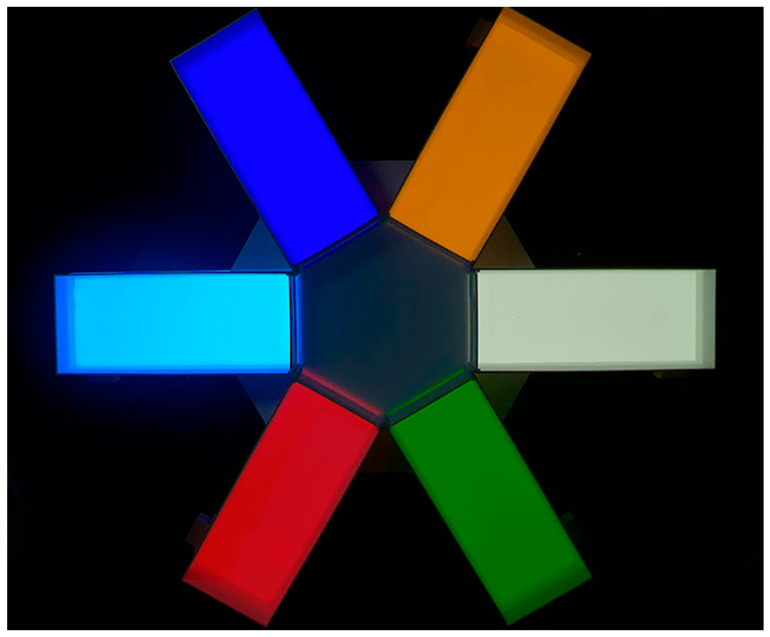
Top view of the experimental apparatus for testing light color selection.

This experiment was carried out in a designed hexagonal maze apparatus consisting of six rectangular cubes (length × width × height = 30 cm × 20 cm × 15 cm) with a square hexagonal prism (side length 20 cm, height 15 cm) (Figure 2). The bottom of each rectangle had a light-transmitting white acrylic sheet, which could be set up with different areas by inserting LED light panels of different light colors at the bottom. The order of light panels was randomly inserted for each trial. A removable black spacer between each rectangle and the hexagonal prism was used to isolate the light from the rectangle. Based on previous research and light colors commonly found in aquaculture [38,39], violet (410–420 nm), yellow (580–590 nm), green (550–560 nm), red (620–630 nm), blue (470–480 nm), and white were selected as light colors. The LED light board was rated at 40 W, the operating voltage was DC 24 V, and the average light intensity in the water was set to 1000 lx by means of a transformer. Fresh aeration water was used as experimental water, and the depth of the water was controlled at 7 cm. Water was changed, and the apparatus was cleaned after each experiment. In each experiment, one fish had to be transferred to the center of the hexagonal prism for 15 min of dark adaptation. Before withdrawing the partition, a 30 min video recording (30 frames/s) was started by using a high-speed industrial camera (acA1920, 155uc NIR, Balser, Germany) located approximately 1.5 m above the apparatus. After the experiment was finished, all fish were released into their source, the Yangtze River.

### 2.3. Data Analysis

In this study, in order to compare personality differences between groups, light color selection behaviors of 12 *P. fulvidraco* juveniles were first analyzed, followed by shy and bold individuals.

A behavior analysis software EthoVision XT 15.0 (Noldus, Wageningen, The Netherlands) was used to automatically analyze fish motion data and to derive activity trajectory heat maps of the experimental fish. For the personality test, the boldness of the fish was quantified by using ImageJ2 (University of Waikato, Hamilton, New Zealand) to calculate the area (cm^2^) of the activity trajectory of the experimental fish (Table 1) [40]. The latency time was used to analyze the exploratory desire and ability. Previous studies have defined different indicators to compare light color preference, including cumulative dwell time and visit frequency [18,34]. Relevant indicators were defined as follows:

Latency time for the first exploration under different areas (s): the time taken from the beginning of the experiment to enter each of the six lights for the first time for the geometric centroid of the experimental fish;

Latency time for full exploration (s): the time taken from the beginning of the experiment to complete its entry into the sixth light for the geometric centroid of the experimental fish;

Visit frequency (times): the number of times the geometric centroid of the experimental fish entered different areas;

Cumulative dwell time (s): the cumulative total time spent by the geometric center of mass of the experimental fish entering and leaving the area of a particular light color multiple times;

Total moved distance (cm): the total distance traveled by the geometric centroid of the experimental fish;

Average speed (cm/s): the average locomotion speed of the geometric centroid of the experimental fish.

In order to more accurately determine the preference of experimental fish for different lights and exclude the influence of bold personalities due to their better exploratory ability on the mean value of experimental data, the percentage of cumulative dwell time and the percentage visit frequency of experimental fish under different lights were chosen as indicators and calculated as follows:

(1) $$P_{t}=t/T\times 100$$

where P_t_ is the percentage of cumulative dwell time (%) of the experimental fish under different lights, t is the cumulative dwell time per fish under different lights, and T is the sum of the cumulative dwell time per fish under the six lights.

(2) $$P_{f}=f/F\times 100$$

where P_f_ is the percentage of visit frequency (%) of experimental fish under different lights, f is the visit frequency per fish under different lights, and F is the sum of the visit frequency per fish under the six lights.

The activity state was divided into three levels, namely lowly active, moderately active, and highly active, based on the temporal change in pixel value across all frames of videos. When the activity level is 0, all pixels are the same; when the activity level is 100%, all pixels are different [41]. Therefore, in this experiment, the thresholds of activity were classified as follows:

- Lowly active: below 20% activity;
- Moderately active: above 20% and below 80% activity;
- Highly active: above 80% activity.

For the percentage of cumulative duration of different active states of the experimental fish under different lights, the following formula was used:

(3) $$P_{c}=c/T\times 100$$

where P_c_ is the percentage of cumulative duration of active state (%) of the experimental fish under different lights, c is the cumulative duration of different active states per fish under different lights, and T is the total cumulative duration of each fish under the six lights.

### 2.4. Statistical Analysis

The experimental raw data were routinely processed and calculated using Microsoft Excel 2022 and subsequently analyzed using the statistical software SPSS 28.0. After testing the normality, one-way analysis of variance (ANOVA) with Tukey’s post hoc test was used to compare differences among the six light colors and three activity states. Independent samples *t*-tests (corrected *t*-tests) were conducted for differences between shy and bold groups. Statistical values are expressed as mean ± standard error (mean ± SE), and *p* < 0.05 was considered statistically significant. Graphs were created using GraphPad Prism 9.5 (La Jolla, San Diego, CA, USA).

## 3. Results

### 3.1. Light Color Selection of Juvenile P. fulvidraco

**Figure 3:**
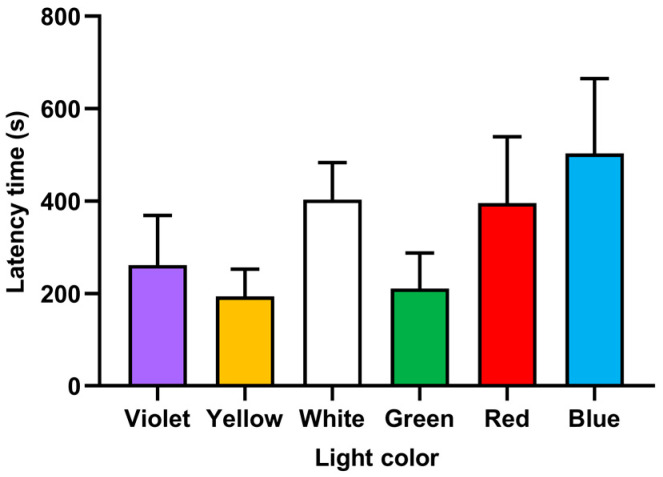
Latency time of fish for the first exploration under six colors of light.

After the black partition was withdrawn, individuals of juvenile *P. fulvidraco* selected the nearest light color area to enter quickly in two seconds or less and then spontaneously explored different lights, showing obvious phototaxis. As shown in Figure 3, there was no significant difference in latency time for the first exploration under the six lights (*F* = 1.25, *p* > 0.05).

**Figure 4:**
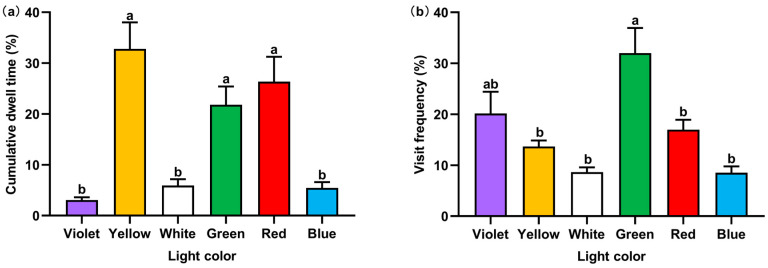
The light color selection of fish: (**a**) cumulative dwell time (%) ${(P}_{t})$; (**b**) visit frequency (%) ($P_{f}$). Different letters (a,b) associated with the bars indicate significant differences (*p* < 0.05).

*P. fulvidraco* juveniles showed an obvious preference in the distribution across violet, yellow, white, green, red, and blue lights (Figure 4a). The average cumulative dwell time was significantly longer with yellow (32.81 ± 5.22%), green (21.81 ± 3.58%), and red lights (26.36 ± 4.89%) compared to violet (3.07 ± 0.55%), white (5.91 ± 1.24%), and blue lights (5.44 ± 1.16%) (*F* = 14.352, *p* < 0.01). Additionally, the average percentage of visit frequency in violet, yellow, white, green, red, and blue lights were 20.15 ± 4.27%, 13.67 ± 1.18%, 8.66 ± 0.90%, 32.00 ± 4.93%, 16.97 ± 1.95%, and 8.54 ± 1.23%, respectively (Figure 4b). The visit frequency also showed a significant difference among the six lights (*F* = 9.27, *p* < 0.01). Overall, for both the cumulative dwell time and visit frequency, juveniles showed a significant preference for the green light.

**Figure 5:**
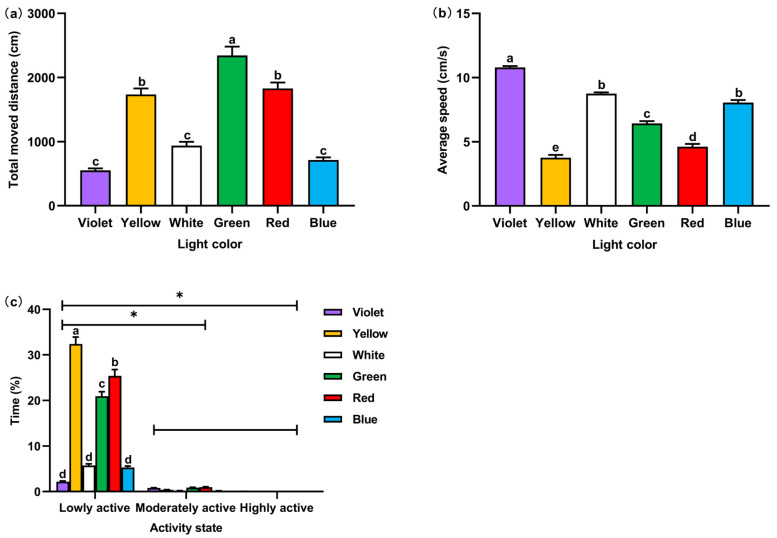
The behaviors of *P. fulvidraco* under six colors of light: (**a**) total traveled distance (cm); (**b**) average speed (cm/s); (**c**) cumulative duration of activity state (%) ($P_{c}$). Different lowercase letters associated with the bars indicate significant differences (*p* < 0.05). An asterisk (*) denotes a significant difference between two activity states (*p* < 0.05).

The total traveled distance of *P. fulvidraco* juveniles was the longest in green light, followed by yellow and red lights, which was significantly higher than that in violet and blue lights (*F* = 73.58, *p* < 0.01) (Figure 5). The average speed of movement had a large variation, showing significant differences under the six colors of light (*F* = 223.49, *p* < 0.01). Individuals of juvenile *P. fulvidraco* in green light had a medium average speed. In violet, white, and blue light, the average speed was higher, and the average speed was lower in the other two lights. As for activity states, regardless of the light colors, *P. fulvidraco* juveniles were mainly in a low active state, showing significant differences (*F* = 8.58, *p* < 0.01), which were consistent with normal swimming conditions.

### 3.2. Personality Analysis of Juvenile P. fulvidraco

**Figure 6:**
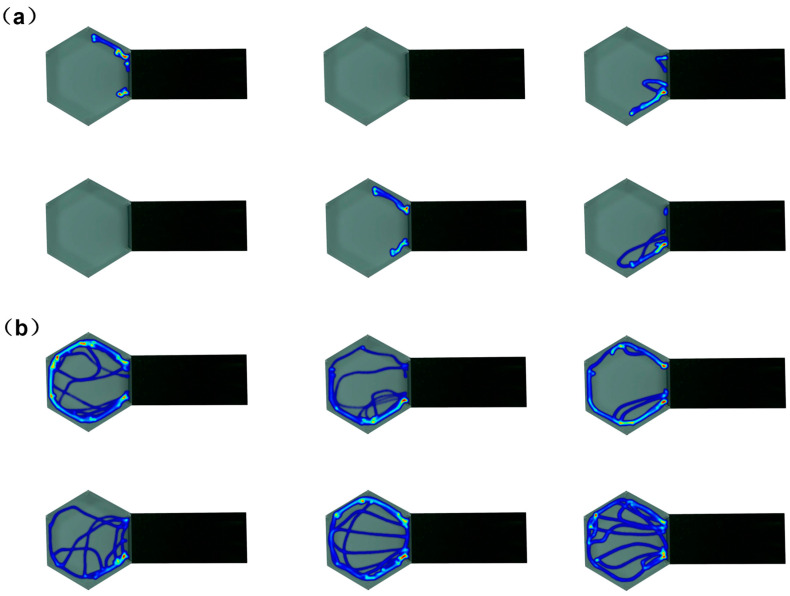
Activity trajectory heat map of different personalities of fish: (**a**) shyness; (**b**) boldness.

The trajectory area of experimental fish was quantified by ImageJ software, and 12 *P. fulvidraco* juveniles with obvious personality, including 6 shy individuals (72.38 ± 24.08 cm^2^) and 6 bold individuals (464.11 ± 42.53 cm^2^) (see Table 1), were analyzed. There were significant differences in their trajectory areas (*p* < 0.05), and the relevant heat map is shown in Figure 6. The total length, body mass, and condition factor were assessed, and the differences were not significant (*p* > 0.05), indicating that the grouping was reasonable.

### 3.3. Light Color Selection of Shy and Bold P. fulvidraco Juveniles

**Figure 7:**
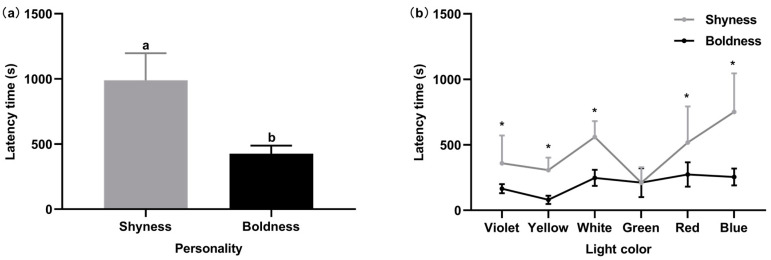
Latency time for full exploration between different personalities (**a**). Latency time for the first exploration between different personalities (**b**). Different lowercase letters associated with the bars indicate significant differences (*p* < 0.05). An asterisk (*) denotes a significant difference between different personalities (*p* < 0.05).

Personalities significantly affected some behaviors of *P. fulvidraco* under the six lights (Figure 7), including the latency time for full exploration and the first exploration. All experimental fish largely finished exploring the six light colors by approximately the 15th minute. However, shy individuals (989.02 ± 208.30 s) had significantly more latency time for full exploration than bold individuals (425.36 ± 62.98 s) (*t*_10_ = 2.59, *p* < 0.05), spending almost twice as much time as bold individuals. In the latency time for the first exploration, shy and bold *P. fulvidraco* showed no significant difference (*p* > 0.05) ingreen light, while they showed a significant difference in the other five lights (*p* < 0.05). They showed a consistent trend of choice for the green light.

**Figure 8:**
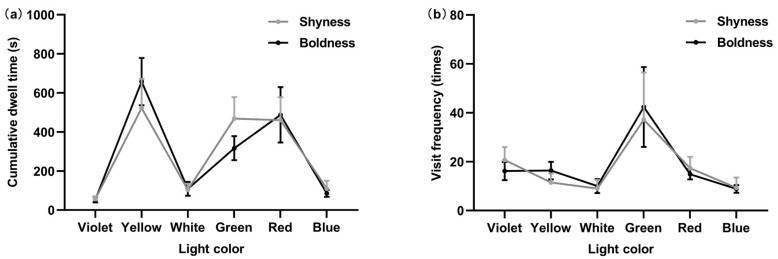
Cumulative dwell time between different personalities under six colors of light (**a**). Visit frequency between different personalities under six colors of light (**b**).

Then, we focused on specific selection behaviors between shy and bold individuals of *P. fulvidraco*. The results of the one-way analysis of variance, shown in Figure 8, indicated that shy and bold *P. fulvidraco* were not distinguishable in cumulative dwell time and visit frequency under the six lights. Different personalities of *P. fulvidraco* were consistent for light color preference.

## 4. Discussion

### 4.1. Light Color Selection of Juvenile P. fulvidraco

*P. fulvidraco* juveniles might prefer the yellow and green lights to the other light colors. In this study, two indicators, namely the cumulative dwell time and visit frequency, were used to analyze the light color preference, and the results are not entirely consistent. *P. fulvidraco* juveniles stayed longer in yellow light and moved in and out of the green light more. In green light, *P. fulvidraco* juveniles had the longest total traveled distance but moved less in yellow light. These two colors may be favored for different reasons. Water absorbance properties of different light colors may cause changes in fish judgments. In violet light, *P. fulvidraco* juveniles had a higher visit frequency, but they showed significant avoidance (short cumulative dwell time). These results suggested that they may have different desires to explore different light colors. No significant difference in latency time for the first exploration was observed under the six lights (Figure 3), which may be due to the small number of test groups.

The above behavioral differences suggested that *P. fulvidraco* juveniles may have the ability to recognize specific colors. The sensitivity of the fish to different colors of light is determined by optochromes in the cone cells, which have different spectral sensitivities, resulting in different phototropism in fish [42,43]. When the partition was removed, the fish quickly entered a nearby light area, which is an important manifestation of phototropism in *P. fulvidraco* juveniles. However, the color vision of *P. fulvidraco* is still unknown, and colors to which they are less sensitive might be not detected. *P. fulvidraco* juveniles were mostly low active under all six lights. They moved longer distance and had lower average speed, which was basically consistent with their random swimming patterns and reflected the fish’s free choice of light.

There are few studies on the phototropic behavior of *P. fulvidraco*. Only Bai et al. reported that *Pelteobagrus vachelli*, the same family as *P. fulvidraco*, is a negatively phototropic fish [34]. This may be due to the fact that the phototropic behavior of fish changes according to the developmental stage [20]. The development stages of fish affect the different phototropic behaviors of fish. Boyd et al. pointed out that the choice of a bright environment by early juvenile fish was associated with the use of vision for feeding [44]. Some benthic fish can change their bait from zooplankton to benthic organisms and thus begin to inhabit, reproduce, and solicit bait on the bottom. The sense of smell becomes the primary feeding sense organ, and vision becomes a secondary sense organ accordingly. At this time, the phototropic behavior weakens or disappears, which also leads to the emergence of negative phototropism [45]. The behavior of organisms is the result of their evolution over time, but the conditions of life chosen by fish have also contributed to the evolution of their behavior [46].

### 4.2. Light Color Selection of Shy and Bold P. fulvidraco Juveniles

By comparing the latency time between the full exploration and initial exploration behaviors of *P. fulvidraco* juveniles with different personalities, we found that the latency time required by bold individuals was about half that of shy individuals, which is consistent with the exploratory ability of bold individuals. At about the fifth minute of the trial, the bold individuals had completed the exploration of the six lights, indicating that bold individuals may have a better ability to adapt when entering a new environment. Fish’s personality fundamentally influences how individuals utilize space. Bold individuals have greater exploratory skills and are able to explore space and utilize resources more quickly [29].

No direct relationship was found between personality and light selection in this work. Their preference for light was consistently identified by comparing the cumulative dwell time and visit frequency between different personalities. Furthermore, regarding the latency time for the first exploration, shy and bold individuals had no significant difference only in green light and showed the same strong desire for the green light. Bold individuals visited all areas quickly, but their total visit frequency was close to that of shy fish. This indicated that when bold fish found preferred lights, they explored less frequently. So, bold fish rapidly decided the preferred light colors, which may depend on the exploration ability.

### 4.3. Future Considerations

As one of the most important environmental factors in aquaculture, light affects fish growth, feeding, and physiological and biochemical indicators [47]. Creating a suitable aquaculture environment can reduce fish stress, promote fish growth, and improve fish nutritional value. Studies have shown that light color is closely related to fish growth and feeding. A red-light environment has been found to enhance the growth of pikeperch (*Sander lucioperca*) [48]. When steelhead trout (*Oncorhynchus mykiss*) are exposed to a blue–red–blue daily rhythm of light color variation, their growth and immunity are improved [39]. So, the color of light could be used to improve animal welfare. According to the above research findings, the culture of *P. fulvidraco* juveniles under yellow or green culture light can be considered to improve its welfare. Meanwhile, we should pay attention to the growth and physiological performance of *P. fulvidraco* juveniles under preferred culture light, which can provide a theoretical basis for its efficient culture. In addition to wavelength, the effect of light intensity, as one of the important indicators of light [49], should also be considered in actual culture. Although this research offers some suggestions about the culture light color selection, it remains a challenge to utilize the natural light from the wild, and the expression of natural behaviors should be focused on.

In practical applications related to fish personality, bold individuals based on fish behavior can be selected to improve the efficiency of culture and domestication by combining studies of personality traits and adaptability. Alternatively, when selecting fish species for stocking, bold individuals can be selected to improve the survival rate. By measuring morphological characteristics, Xiang et al. found that bold and shy fish have a significant difference in appearance [50]. This method may not be applicable to all fish. If we had a way to characterize bold fish more quickly, it would help us understand fish behavior better, and thus more research on quantifying fish appearance of different personalities could be conducted. Although no difference in light color preference was found in this study among the different personalities, it is crucial to pay attention to the adaptive capacity of individuals in animal welfare.

## 5. Conclusions

In the present study, *Pelteobagrus fulvidraco* juveniles showed different selective behaviors for six light colors: violet (410–420 nm), yellow (580–590 nm), green (550–560 nm), red (620–630 nm), blue (470–480 nm), and white. Among the six lights, the yellow and green lights may be favored for different reasons. Our results are helpful to the welfare of *P. fulvidraco* juveniles. However, it will be a long-term challenge to further explore the growth and physiological condition of fish under culture lights. The results from this work also indicated that shy and bold fish have the same preference for the green light. Bold individuals could find the preferred light colors quickly and makequick decisions for light color selection. After identifying the preferred light colors, they reduced the frequency of exploration and then stayed more in their preferred lights. Thus, more refined work should be undertaken to quantify the characteristics of bold fish.

## Figures and Tables

**Table 1:** The activity areas and growth of bold and shy *P. fulvidraco* juveniles used in light exposure experiment.

	Personality		Levine Variance Isotropy Test		Independent-Samples *t*-Test	
	Shy	Bold	F	P	df	Sig. (Two-Tailed)
Area (cm^2^)	72.38 ± 24.08	464.11 ± 42.53	10.185	0.01	10	0.00 *
Total length (cm)	10.73 ± 0.22	10.63 ± 0.17	0.07	0.80	10	0.73
Body mass (g)	10.65 ± 0.60	10.43 ± 0.65	0.05	0.82	10	0.81
Condition factor	0.86 ± 0.02	0.86 ± 0.02	0.00	0.97	10	0.88

Notes: “*” means a significant difference (*p* < 0.05).

## Data Availability

The data presented in this study are available on request from the corresponding author.
